# Smartphone-generated 3D facial images: reliable for routine assessment of the oronasal region of patients with cleft or mere convenience? A validation study

**DOI:** 10.1186/s12903-024-05280-9

**Published:** 2024-12-19

**Authors:** Pradeep Singh, Richard Tai‑Chiu Hsung, Deepal Haresh Ajmera, Noha A. Said, Yiu Yan Leung, Colman McGrath, Min Gu

**Affiliations:** 1 Division of Paediatric Dentistry and Orthodontics, Faculty of Dentistry, The University of Hong Kong, Hong Kong SAR, China; 2 Department of Computer Science, Hong Kong Chu Hai College, Hong Kong SAR, China; 3 Discipline of Oral and Maxillofacial Surgery, Faculty of Dentistry, the University of Hong Kong, Hong Kong SAR, China; 4 Discipline of Applied Oral Sciences & Community Dental Care, Faculty of Dentistry, the University of Hong Kong, Hong Kong SAR, China

**Keywords:** Smartphone, 3D, Direct anthropometry, 3dMD, Bellus3D, 3D surface-imaging, Cleft, Oronasal

## Abstract

**Objectives:**

To evaluate the validity and reliability of smartphone-generated three-dimensional (3D) facial images for routine evaluation of the oronasal region of patients with cleft by comparing their accuracy to that of direct anthropometry (DA) and 3dMD.

**Materials and methods:**

Eighteen soft-tissue facial landmarks were manually labelled on each of the 17 (9 males and 8 females; mean age 23.3 ± 5.4 years) cleft lip and palate (CLP) patients’ faces. Two surface imaging systems, 3dMDface and Bellus3D FaceApp, were used to perform two imaging operations on each labelled face. Subsequently, 32 inter-landmark facial measurements were directly measured on the labelled faces and digitally measured on the 3D facial images. Statistical comparisons were made between smartphone-generated 3D facial images (SGI), DA, and 3dMD measurements.

**Results:**

The SGI measurements were slightly higher than those from DA and 3dMD, but the mean differences between inter-landmark measurements were not statistically significant across all three methods. In terms of clinical acceptability, 16% and 59% of measures showed differences of ≤ 3 mm or ≤ 5º, with good agreement between DA and SGI and 3dMD and SGI, respectively. A small systematic bias of ± 0.2 mm was observed generally among the three methods. Additionally, the mean absolute difference between the DA and SGI methods was the highest for linear measurements (1.31 ± 0.34 mm) and angular measurements (4.11 ± 0.76º).

**Conclusions:**

SGI displayed fair trueness compared to DA and 3dMD. It exhibited high accuracy in the orolabial area and specific central and flat areas within the oronasal region. Notwithstanding this, it has limited clinical applicability for assessing the entire oronasal region of patients with CLP. From a clinical application perspective, SGI should accurately encompass the entire oronasal region for optimal clinical use.

**Clinical relevance:**

SGI can be considered for macroscopic oronasal analysis or for patient education where accuracy within 3 mm and 5º may not be critical.

**Supplementary Information:**

The online version contains supplementary material available at 10.1186/s12903-024-05280-9.

## Introduction

People with Cleft lip and palate (CLP) exhibit distinct facial characteristics, and the oronasal region is particularly affected, with the severity of the cleft determining the extent of the impact [1]. To effectively diagnose and rehabilitate CLP deformities, a thorough investigation of the oronasal morphology is essential. The treatment of CLP cases necessitates meticulous planning, with imaging playing a pivotal role. Traditional two-dimensional (2D) methods such as 2D photos [2], Vernier callipers, and a bevel protractor [3, 4] have intrinsic limits encompassing facial depth, form, area, and volumetric measurements [4–7]. Consequently, three-dimensional (3D) face acquisition has gained popularity [5–10] and demonstrated significant advancements over traditional 2D methods, leading to enhanced diagnostics, treatment planning, and surgical outcomes in the realm of craniofacial research and practice. Presently, 3D surface-imaging technologies not only offer more comprehensive information and eliminate ionizing radiation associated with conventional imaging methods [11], but also exhibit commendable attributes of high precision, accuracy, non-invasiveness, and rapid acquisition [12, 13]. Moreover, these technologies facilitate rotation and analysis of 3D images, enable digital recording of facial landmarks, and aid in tracking pre- and post-operative changes. Additionally, 3D surface imaging's capacity to record, replicate, and model the anatomy of the face has been shown to be an effective perioperative tool for evaluating surgical results and acquiring intricate information concerning craniofacial structures for orthodontics and cranio-maxillofacial surgery purposes [7, 14, 15] including planning, capturing facial emotions, and facial recognition [15–18]. Therefore, given the prolonged treatment time for cleft and craniofacial care, the utilisation of 3D surface imaging holds significant promise as a beneficial tool for diagnosis, planning, audit, and long-term evaluation of post-operative outcomes and is already being employed in cleft lip and palate clinics across the globe.

The use of 3D imaging has emerged as a contemporary approach in cleft care, offering a proficient means to capture the morphology of the oronasal complex and quantitatively assess oronasal attributes in patients with CLP [1, 19–24]. The utilization of 3D facial images is widely acknowledged as the most reliable tool for detecting, planning, and predicting treatment results [6, 7, 25]. Indeed, it has been recommended as a customary practice for capturing the oronasal region of patients with CLP [26]. As a result, a handful of scientific publications have employed intraoral scanners to study the nasolabial region [27, 28], with *Olmos *et al*.’s* confirming the efficacy of these scanners in capturing the nasolabial region in CLP models [27] and *Ayoub *et al*.* validating their application for assessing lip asymmetry and scarring in patients with CLP [28]. In addition, other advanced 3D surface-imaging technologies, such as stereophotogrammetry, laser-based scanning, and structured light scanning, have been devised to capture highly realistic 3D facial images. Nevertheless, their practical implementation in routine clinical environments is currently limited due to their exorbitant cost, the need for skilled personnel, a designated area for stationary cameras, and robust computer systems to handle image processing [29, 30]. In order to address these practical challenges, there is an increasing interest in leveraging mobile phone technology for capturing 3D facial images in numerous medical and dentistry fields [31, 32]. Consequently, the employment of smartphones for capturing 3D facial data is becoming increasingly popular due to their advantages of being rapid, easy to use, portable, and cost-effective. Furthermore, this approach permits image processing, storage, and subsequent dissemination, thereby enabling a portable alternative for the acquisition of clinically acceptable 3D facial data.

Although prior studies have examined the use of smartphone-based 3D face acquisition in facial morphology research [33–39], there are no studies on the application of smartphone-generated 3D facial images (SGI) for analyzing oronasal morphology in patients with CLP. Additionally, there is a dearth of information about the validity of SGI, with inconsistent accuracy reported in the previous investigations [3, 14, 40, 41]. While some research encouraged the clinical application of smartphone photogrammetry, reporting an accuracy of 1.2 mm to 1.3 mm using an iPhone against the gold standard, the Artec Spider light scanner [14, 41], others reported conflicting results [3, 40]. Furthermore, for optimal analysis and 3D treatment planning of the oronasal region, which comprises the nose, lips, and adjacent soft tissue landmarks, it is imperative that the 3D facial image exhibit clinically acceptable precision in 3D. The aforementioned encompasses accuracy in the central to lateral oronasal, as well as from frontal to lateral views. Consequently, despite the potential to be a low-cost and practical alternative for 3D face acquisition, SGI has not been employed for studying oronasal morphology in CLP cases, and the validity of SGI for clinical usage in patients with CLP remains uncertain. Therefore, the present study aimed to investigate the validity of SGI for the routine assessment of the oronasal region in patients with CLP. The primary objective was to objectively compare the accuracy of SGI to that of direct anthropometry (DA) and 3dMD, which are both considered to be gold standards for photogrammetry [1, 42]. We believe that this comparison will reveal any potential disparities between SGI and the gold standards, thereby providing a true estimate of SGI’s accuracy. The null hypothesis was that there would be no noticeable difference between the measures acquired from SGI and those obtained from DA and 3dMD. To our knowledge, this is the first study to assess the accuracy of SGI specifically in the oronasal region, encompassing the nasal, nasolabial and orolabial areas, by comparing SGI with DA and 3dMD.

## Materials and methods

### Study design

This prospective experimental study intended to validate the accuracy of SGI for routine clinical use in patients with cleft. To achieve so, the linear and angular measurements obtained from SGI of patients with CLP were compared to those obtained from DA and 3dMD-generated images of the same patients.

### Sampling and sample

For this study, a sample of 17 patients with CLP was recruited from the orthodontic-orthognathic patient pool of the Prince Philip Dental Hospital, University of Hong Kong, between December 2020 and March 2021. The sample consisted of 9 males and 8 females, with a mean age of 23.3 ± 5.4 years. The inclusion criteria were as follows: (1) Chinese subjects (similar ethnicity); (2) individuals who had undergone repair for cleft lip (CL) or CLP; (3) age > 18 years; (4) non-syndromic CL or CLP patients; and (5) no history of facial surgery. Subjects with a cleft palate, alveolus, or soft palate exclusively, as well as those with unclear 3D images, were excluded from the study.

Based on a previous study and using the intraclass correlation coefficient (ICC) to define a substantial agreement of > 0.8, with a power of 80% and a significance level of 5% (two-sided), a minimum sample of 13 participants was determined to be necessary [43]. To account for a potential drop-out rate of 15%, a total of 17 participants were recruited for the study.

### Landmark annotation

**Fig. 1:**
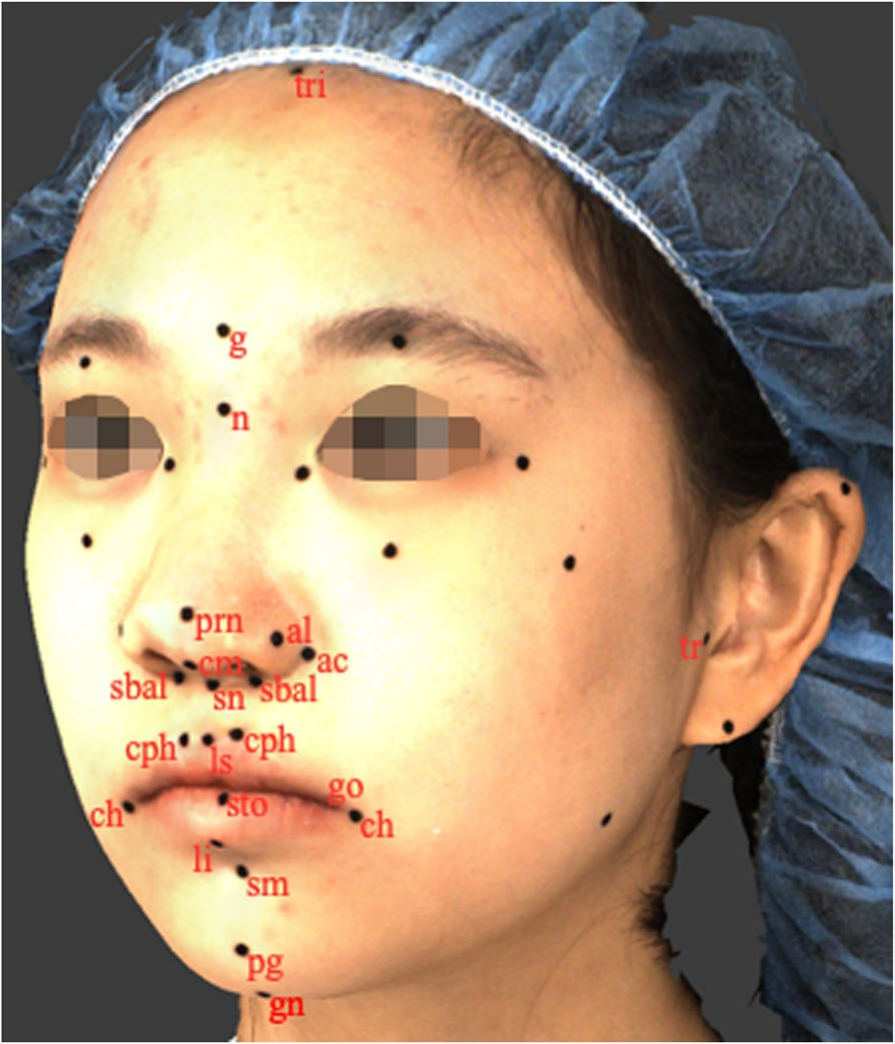
Anthropometric landmarks: *tri*, trichion; *g*, glabella; *n,* nasion; *tr,* tragion; *gn,* gnathion; *prn,* pronasale; *sn,* subnasale; *al,* alare; *ac,* alar crest; *sbal*, subalare; *cm,* columella; *ls,* labiale superius; *li,* labiale inferius; *sto,* stomion; *cph,* crista philtri; *ch,* cheilion; *pg,* pogonion; *sm,* supramental

A total of 18 anthropometric soft-tissue facial landmarks, which had been previously defined in the literature [1, 44–48], were manually identified and labelled on the patient's face using black round adhesive stickers with a diameter of 2 mm (Fig. 1). The specific landmarks used in this study are listed in Table 1.

Anthropometric landmarks and their definition

**Table 1:** Anthropometric landmarks and their definition

**Landmark**	**Abbreviation**	**Definition**
***Trichion***	tri	Point on the hairline in the midline of the forehead
***Glabella***	g	Most prominent midline point between the eyebrows
***Nasion***	n	Point in the midline of both the nasal root and the nasofrontal suture
***Tragion *** **(BL)**	tr	Notch on the upper margin of the tragus
***Gnathion***	gn	Lowest median landmark on the lower border of mandible
***Pronasale***	prn	Most protruded point of the apex nasi in lateral view
***Subnasale***	sn	Midpoint of the angle at the columella base where the lower border of the nasal septum and surface of the upper lip meet
***Alare *** **(BL)**	al	The most lateral point on each alar contour
***Alar crest *** **(BL)**	ac	Most lateral point in the curved base line of the ala
***Subalare *** **(BL)**	sbal	Point at the lower limit of alar base
***Columella***	cm	Point on the lower surface of the nose
***Labiale superius***	ls	Midpoint of the upper vermillion border
***Labiale inferius***	li	Midpoint of the lower vermillion line
***Stomion***	sto	Imaginary point at the crossing of the vertical facial midline and the horizontal labial fissure between gently closed lips
***Crista philtri *** **(BL)**	cph	Point on elevated margin of the philtrum just above the vermilion border
***Cheilion *** **(BL)**	ch	Lateral limit of each labial commissure
***Pogonion***	pg	Most anterior midpoint of the chin
***Supramental***	sm	The deepest point of the inferior sublabial concavity

*BL* Bilateral

### 3D Facial image acquisition

**Fig. 2:**
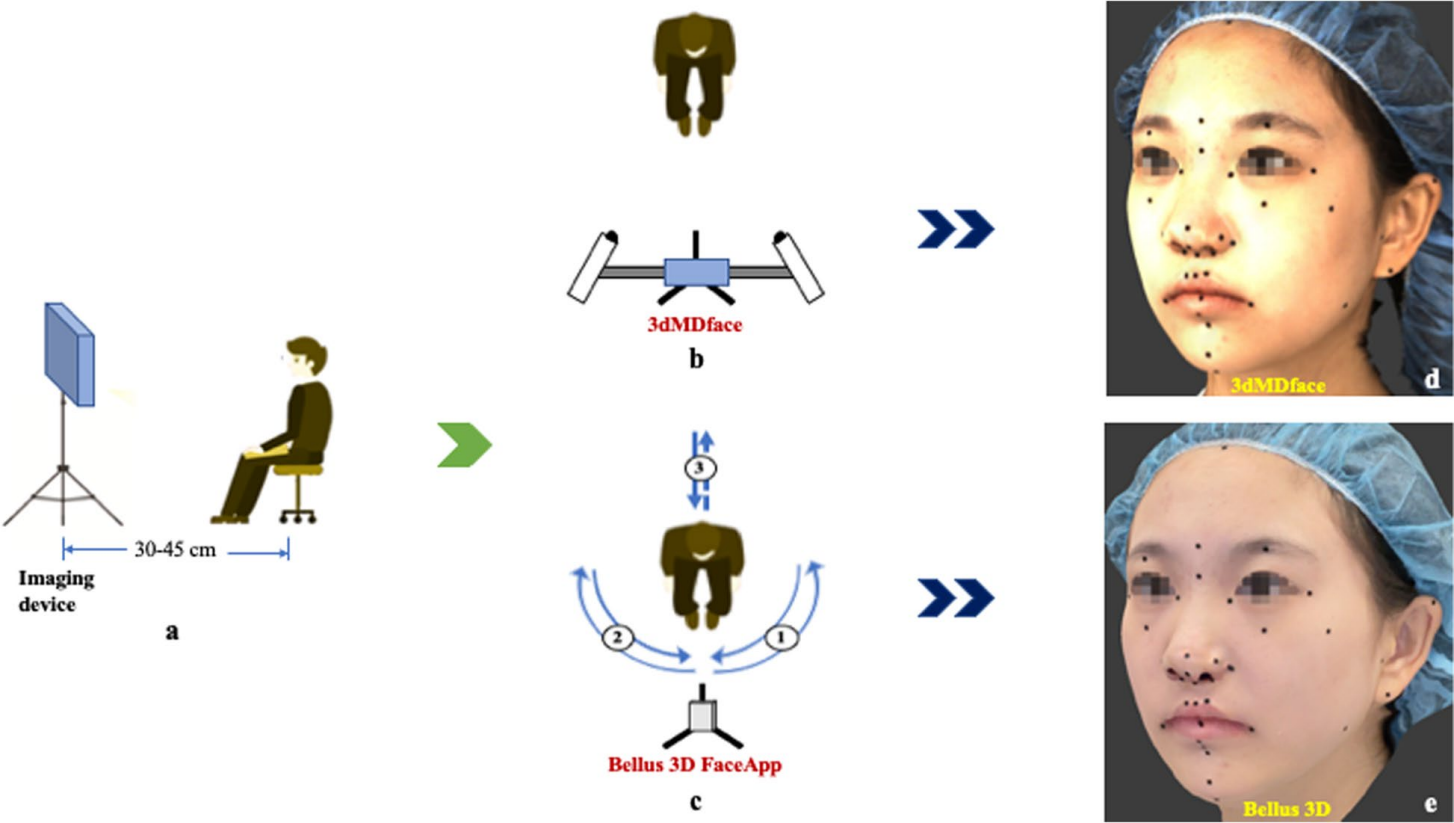
Schematic representation of imaging operations: **a** Imaging set-up; **b** Facial image acquisition using 3dMDface; **c** Facial image acquisition using Bellus3D FaceApp; **d** Three-dimensional facial image generated using 3dMDface; **e** Three-dimensional facial image generated using Bellus3D FaceApp

Each participant in the study was instructed to sit upright and adopt a natural head position (NHP) [49]. They were asked to keep their eyes wide open and maintain minimal facial expression and maximum intercuspation position (MIP). To ensure standardized imaging conditions, participants were seated on a comfortable adjustable chair at a distance of 30–45 cm from the imaging device in a room with 10,000 lx and 4100 K illuminance, with no windows (Fig. 2a). The imaging procedures were conducted in high-definition (HD) mode by the same operator in the same room. Before capturing the images, participants were required to remove any accessories that could affect image capture, such as earrings, necklaces, or glasses. They were also asked to wear a standardized head cap to expose the entire facial skin, including the forehead and ears [50]. Calibration was performed according to the manufacturer's guidelines as an initial step in the image acquisition process. For each labelled face, two imaging operations were conducted utilizing two separate surface imaging systems. The first system employed was the *3dMDface system* (3dMD LLC, Atlanta, GA, USA; https://3dmd.com/), which captured the object's surface by simultaneously taking photos from multiple angles with millisecond precision. This system utilized machine vision cameras, an infrared pattern projector, and light-emitting diode (LED) lighting to generate high-quality 3D images (Fig. 2b). The second system employed was the *Bellus3D FaceApp* (version 3P; Bellus3D, Inc., Campbell, CA, USA; https://www.bellus3d.com), a free-to-use face scanning mobile application (app) for iPhones that utilized the iPhone's built-in TrueDepth camera for image acquisition. The smartphone was mounted on a tripod, and participants were instructed to rotate their heads as directed by the app's graphic interface and voice instructions while maintaining the NHP (Fig. 2c). After capturing the images, they were reconstructed (Fig. 2d and e) using the associated software programmes (3dMD and *Bellus3D FaceApp*, respectively) and exported in OBJ (object file) file format. Following the image acquisition step, participants were prepared and instructed for the ensuing measuring procedure.

### Measurements

**Fig. 3:**
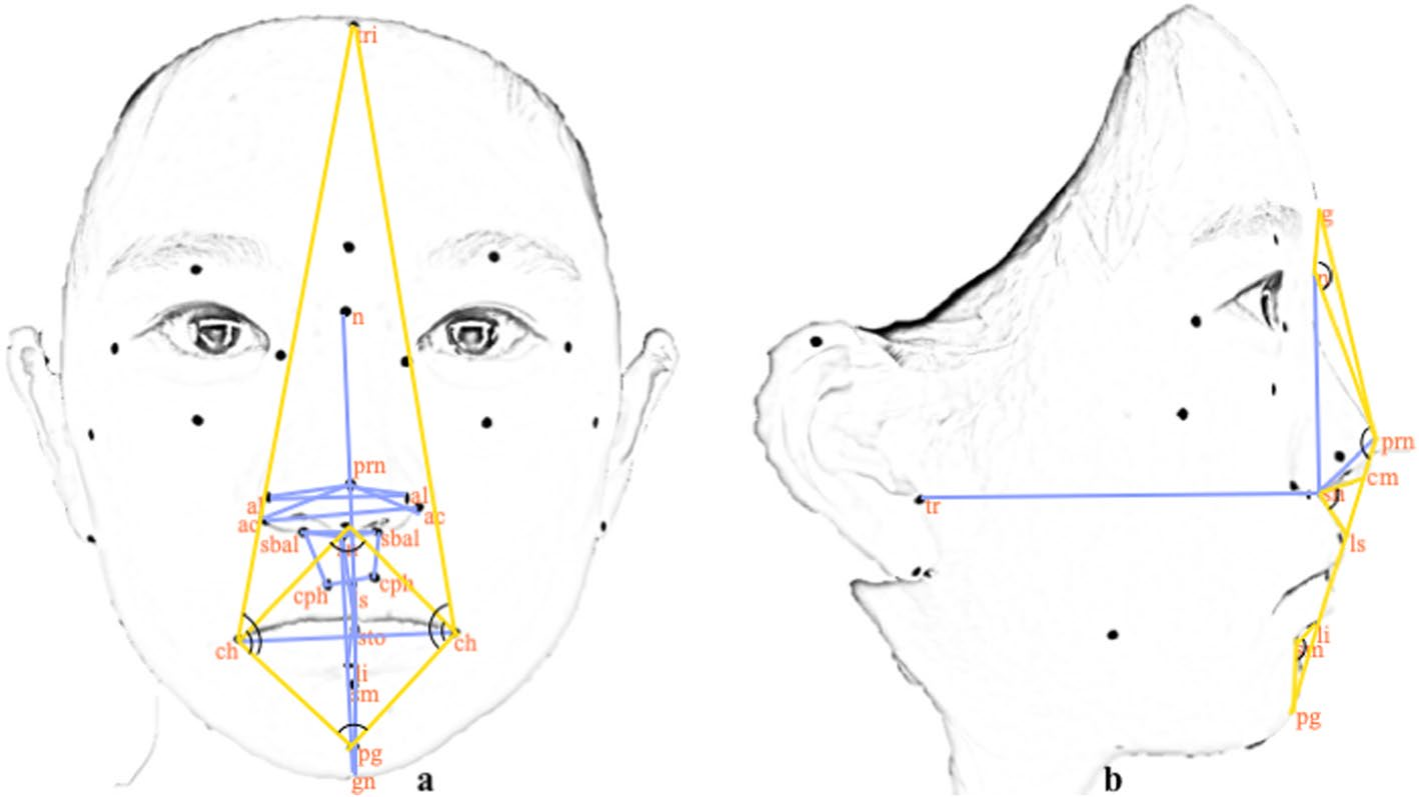
Schematic depiction of linear (in blue) and angular (in yellow) inter-landmark measurements in the frontal (**a**) and lateral views (**b**)

The study utilized a comprehensive set of linear and angular measurements, as outlined in Table 2. A total of 32 inter-landmark measurements were performed, including 22 linear measurements (19 in frontal view and three in lateral view) and 10 angular measurements (six in frontal view and four in lateral view) among the identified facial landmarks (Fig. 3a and b). These measurements were obtained by directly measuring each annotated face and digitally measuring 3D facial images using *DI3Dview*, a specialized 3D mesh-processing software programme (Dimensional Imaging, Glasgow, Scotland). To ensure consistency, participants were instructed to retain the same seating position and facial expression during direct measurements as specified while capturing 3D facial images.

**Table 2:** Description of linear and angular measurements

Measurements	Annotation	Description	Image view
***Linear***	al_al	Nose width (inter-alar distance)	F
	al_prn	Pronasale to alar base	F
	ac_ac	Anatomical width of the nose	F
	ac_prn	Length of the ala	F
	sbal_sbal	Subalare width	F
	sbal_sn	Subalare to subnasale	F
	sbal_cph	﻿Upper lip lateral length	F
	cph_cph	Width of philtrum	F
	ls_sto	Vermilion height of the upper lip	F
	sto_li	Vermilion height of the lower lip	F
	sn_sto	Height of the upper lip	F
	sto_gn	Height of the mandible	F
	ch_ch	Width of the mouth/ length of labial fissure	F
	n_sto	Height of the upper face	F
	sn_gn	Height of the lower face	F
	tr_sn	Depth of middle third of the face	L
	n_sn	Nose height	L
	sn_prn	Nasal tip protrusion	L
***Angular***	∠tri_ch_pg	Angle formed by trichion, chelion and pogonion	F
	∠ch_sn_ch	Angle formed by right chelion, subnasale and left chelion	F
	∠ch_pg_ch	Angle formed by right chelion, pogonion and left chelion	F
	∠sn_ch_pg	Angle formed by subnasale, chelion and pogonion	F
	∠g_n_prn	Nasofrontal angle	L
	∠g_prn_pg	Total facial convexity	L
	∠cm_sn_ls	Nasolabial angle	L
	∠li_sm_pg	Mentolabial angle	L

*F* Frontal, *L* Lateral

For DA, linear measurements were obtained with a Vernier calliper (VINCA DCLA-0605, Clockwise Tools Inc., Valencia, CA, USA) accurate to 0.01 mm, and angular measurements were acquired with a digital protractor (iGaging, California, USA). To safeguard the soft tissue integrity, the measuring tip of the Vernier calliper and the digital protractor were lightly placed on the stickers without applying pressure [51].

### Outcome measures

To quantitatively analyse the measures, the measurements acquired from SGI were compared with those from DA and 3dMD, which were considered the "reference values." The validity of SGI was stated as a measure of accuracy, which was established by the capacity of the imaging system to capture the participant’s oronasal characteristics accurately with minimum measurement error compared to the reference values. Additionally, 3dMD measurements were directly evaluated against DA values for further analysis.

### Error study

A single examiner (PS), who was trained and experienced, conducted all the measures. To analyse the intra-examiner reliability and method error, the same examiner recorded all the digital measures once again after a washout period of 2 weeks.

### Statistical analysis

The collected data was analysed using IBM Statistical Package for the Social Sciences (SPSS) version 25.0 (SPSS for Mac, IBM Corp., Armonk, N.Y., USA). Intra-examiner reliability was examined using the intra-class correlation coefficient (ICC), where a value close to 1 indicated high reliability and a value close to 0 indicated low reliability [52]. To determine the method error, Dahlberg's formula was utilised [53]. The normality of the data distribution was verified using the Shapiro–Wilk test. To evaluate the accuracy of SGI, multiple error magnitude statistics were used. Accuracy was measured in terms of mean, standard deviation (SD), and mean absolute difference (MAD), which is the average absolute difference between the reference values and SGI measurements. A one-way analysis of variance (ANOVA) was employed to compare the difference in the means between the three methods (DA, 3dMD and SGI).

To assess the agreement between different methods, Bland–Altman analyses were conducted [54]. In this analysis, a total deviation of ± 3.0 mm for linear measurements [39] and ± 5º for angular measurements [55] was considered clinically acceptable. Therefore, any 95% limit of agreement beyond 3 mm and 5º was considered clinically unacceptable. Method validity was evaluated by comparing mean directional differences (DD), standardised directional differences (SDD), and absolute differences (AD) between them. Systematic bias between the groups was tested by calculating the mean DD, taking into account positive and negative signs, and comparing it to zero using a one-sample Student's t-test. Additionally, to estimate the effect magnitude, SDD [56] was derived by dividing DD by the standard deviation (SD) of digital measurements (SDD = DD / SD_digital measurements_). SDD was classified as small if near ± 0.2, medium if close to ± 0.5, and large if close to ± 0.8 or above [57]. Furthermore, MAD was calculated to compare the trueness values of the three methods. To limit the likelihood of falsely rejecting the null hypotheses, the statistical interference of multiple comparisons was adjusted using Bonferroni correction (*p* < 0.05/number of tests), and a significance level of *p* < 0.002 (0.05/32) was considered statistically significant.

## Results

### Reliability assessments

The results of the intra-examiner reliability and method error analysis for each inter-landmark measurement can be found in Supplementary Appendix 1. The intra-examiner reliability was found to be excellent for all the measurements, with a mean ICC of 0.99 (range: 0.95 to 1.00) for both SGI and 3dMD. The method error for linear measurements ranged from 0.04 to 0.14 mm for SGI and 0.04 to 0.29 mm for 3dMD, while for angular measurements it ranged from 0.03 to 0.21º for SGI and 0.02 to 0.22º for 3dMD.

Table 3 presents a comparison of the mean and standard deviation (SD) for each variable between the DA and 3D digital measurements. The mean values of all the linear and angular inter-landmark measurements acquired from SGI were determined to be statistically similar (*p* > 0.002) to measurements from DA and 3dMD.

**Table 3:** Comparison of inter-landmark measurements between SGI, 3dMD and DA

		DA	Digital measurements		
			**SGI**	**3dMD**	
Measurements (mm, °)		Mean ± SD	Mean ± SD	Mean ± SD	*p* ^*#*^
**Linear**	al_al	39.29 ± 4.45	38.09 ± 4.22	39.49 ± 4.29	all *p* values > 0.002
	al_prn_L	24.25 ± 2.80	23.06 ± 3.02	23.46 ± 2.80	
	al_prn_R	24.00 ± 2.95	22.60 ± 2.89	23.52 ± 3.01	
	ac_ac	43.22 ± 4.72	43.86 ± 4.68	44.55 ± 4.92	
	ac_prn_L	29.81 ± 3.46	30.01 ± 3.78	30.76 ± 3.42	
	ac_prn_R	30.19 ± 3.19	29.44 ± 3.31	30.86 ± 3.52	
	sbal_sbal	21.17 ± 4.22	21.15 ± 4.29	21.26 ± 4.16	
	sbal_sn_L	11.44 ± 2.72	11.61 ± 2.77	11.41 ± 2.76	
	sbal_sn_R	10.89 ± 2.70	10.77 ± 2.54	10.84 ± 2.43	
	sbal_cph_L	12.91 ± 2.10	12.10 ± 1.82	12.93 ± 1.88	
	sbal_cph_R	13.35 ± 2.97	12.49 ± 2.76	13.85 ± 2.35	
	cph_cph	13.75 ± 2.99	13.56 ± 2.90	13.70 ± 2.74	
	ls_sto	9.72 ± 2.68	9.65 ± 3.12	10.63 ± 3.06	
	sto_li	10.47 ± 2.29	10.63 ± 2.83	10.28 ± 2.49	
	sn-sto	20.29 ± 3.29	19.18 ± 3.42	20.55 ± 3.34	
	sto_gn	45.51 ± 3.13	47.13 ± 3.27	46.14 ± 3.49	
	ch_ch	54.39 ± 5.55	53.61 ± 6.38	54.31 ± 5.78	
	n_sto	73.91 ± 7.66	74.89 ± 8.27	75.59 ± 8.64	
	sn_gn	64.88 ± 4.15	65.69 ± 4.54	65.21 ± 4.33	
	tr_sn	113.94 ± 8.63	114.68 ± 9.20	113.74 ± 9.24	
	n_sn	54.48 ± 6.42	56.01 ± 6.65	55.81 ± 7.12	
	sn_prn	19.71 ± 2.68	19.48 ± 2.70	20.50 ± 2.61	
**Angular**	∠tri_ch_pg_L	125.66 ± 5.36	129.54 ± 5.24	128.58 ± 5.29	
	∠tri_ch_pg_R	123.11 ± 5.46	126.13 ± 7.03	125.12 ± 6.47	
	∠ch_sn_ch	91.79 ± 6.62	96.28 ± 7.48	95.24 ± 7.10	
	∠ch_pg_ch	76.65 ± 8.97	79.15 ± 8.16	80.28 ± 7.50	
	∠sn_ch_pg_L	83.86 ± 8.62	86.67 ± 7.14	86.67 ± 6.52	
	∠sn_ch_pg_R	83.80 ± 9.74	85.86 ± 9.14	85.38 ± 8.73	
	∠g_n_prn	148.38 ± 8.84	151.53 ± 9.03	151.62 ± 8.85	
	∠g_prn_pg	153.58 ± 4.78	154.07 ± 3.35	153.97 ± 3.64	
	∠cm_sn_ls	93.54 ± 11.59	99.36 ± 11.47	98.04 ± 12.08	
	∠li_sm_pg	132.64 ± 9.23	137.59 ± 8.81	137.61 ± 8.52	

*DA* Direct Anthropometry, *SGI* Smartphone generated 3D facial image, *mm* millimeter, ° Degrees, *SD* Standard Deviation

^#^One-way Analysis of Variance (ANOVA), *p* < 0.002, considered statistically significant

### Method validity (Agreement between Methods)

Table 4 provides a quantification of the Bland–Altman 95% limits of agreement between different methods. In terms of clinically acceptable differences (≤ 3 mm or ≤ 5º), 16% of the measures exhibited good agreement between DA and SGI (*p* > 0.002) for differences that were clinically acceptable when assessing the 95% limits of agreement. Similarly, a significant proportion of measurements, specifically 59% and 38%, demonstrated clinically acceptable differences and good agreement between 3dMD and SGI (*p* > 0.002) and between DA and 3dMD (*p* > 0.002), respectively, when evaluated based on the 95% limits of agreement.

**Table 4:** Bland-Altman analysis for the quantification of agreement between different methods

		Bland-Altman														
		**DA-SGI**					**3dMD-SGI**					**DA-3dMD**				
		95% limits of agreement					95% limits of agreement					95% limits of agreement				
Measurements (mm, °)		**Mean bias**	**SD**	**LL**	**UL**	p*	**Mean bias**	**SD**	**LL**	**UL**	p*	**Mean bias**	**SD**	**LL**	**UL**	p*
Linear	al_al	1.20	1.14	−1.04	3.44		1.40	0.93	−0.43	3.23	all *p* values > 0.002	−0.20	0.86	−1.88	1.48	
	al_prn_L	1.18	1.13	−1.03	3.39		0.40	0.79	−1.16	1.96		0.78	0.94	−1.07	2.63	
	al_prn_R	1.40	1.17	−0.89	3.69		0.92	1.24	−1.50	3.35		0.48	0.61	−0.72	1.68	
	ac_ac	−0.64	1.67	−3.92	2.64		0.68	1.22	−1.71	3.07		−1.32	1.28	−3.82	1.18	
	ac_prn_L	−0.20	1.52	−3.19	2.79		0.75	0.91	−1.02	2.53		−0.95	1.55	−4.00	2.10	
	ac_prn_R	0.75	1.15	−1.51	3.00		1.42	1.22	−0.97	3.81		−0.67	1.48	−3.58	2.24	
	sbal_sbal	0.01	1.05	−2.05	2.08		0.09	1.02	−1.90	2.09		−0.08	1.20	−2.43	2.27	
	sbal_sn_L	−0.15	1.47	−3.04	2.74		−0.18	0.82	−1.79	1.43		0.02	1.28	−2.49	2.54	
	sbal_sn_R	0.12	1.33	−2.49	2.72		0.07	0.55	−1.02	1.16		0.05	1.21	−2.32	2.41	
	sbal_cph_L	0.71	1.27	−1.77	3.20		0.74	1.20	−1.62	3.09		−0.02	1.29	−2.55	2.50	
	sbal_cph_R	0.86	1.48	−2.05	3.76		1.36	0.91	−0.43	3.14		−0.50	1.70	−3.83	2.83	
	cph_cph	0.19	0.87	−1.52	1.90		0.14	0.73	−1.29	1.57		0.05	1.02	−1.96	2.06	
	ls_sto	0.07	1.51	−2.88	3.03		0.98	1.11	−1.19	3.16		−0.91	1.58	−4.01	2.19	
	sto_li	−0.16	1.04	−2.20	1.87		−0.35	0.96	−2.23	1.53		0.18	0.70	−1.20	1.56	
	sn_sto	1.10	1.47	−1.78	3.99		1.37	0.91	−0.41	3.15		−0.27	1.29	−2.80	2.26	
	sto_gn	−1.52	1.64	−4.74	1.69		−0.94	1.23	−3.35	1.48		−0.59	1.48	−3.50	2.32	
	ch_ch	0.78	1.74	−2.63	4.19		0.69	1.41	−2.06	3.45		0.08	1.36	−2.58	2.75	
	n_sto	−0.97	1.87	−4.65	2.70		0.71	1.07	−1.38	2.79		−1.68	1.97	−5.53	2.17	
	sn_gn	−0.81	2.04	−4.82	3.19		−0.49	1.13	−2.71	1.73		−0.32	1.98	−4.21	3.56	
	tr_sn	−0.70	1.81	−4.25	2.85		−0.89	1.54	−3.91	2.13		0.19	1.30	−2.36	2.74	
	n_sn	−1.54	1.66	−4.78	1.71		−0.20	1.28	−2.71	2.31		−1.34	1.42	−4.13	1.46	
	sn_prn	0.23	1.72	−3.15	3.61		1.02	1.02	−0.98	3.01		−0.79	1.30	−3.33	1.76	
Angular	∠tri_ch_pg_L	−3.66	2.18	−7.93	0.62		−0.91	1.16	−3.17	1.36		−2.75	2.45	−7.56	2.06	
	∠tri_ch_pg_R	−2.84	3.17	−9.05	3.36		−0.95	1.92	−4.73	2.82		−1.89	2.97	−7.72	3.93	
	∠ch_sn_ch	−4.49	2.12	−8.65	−0.33		−1.04	1.98	−4.92	2.83		−3.45	2.46	−8.28	1.37	
	∠ch_pg_ch	−2.50	3.91	−10.16	5.16		1.12	2.07	−2.93	5.18		−3.62	3.15	−9.79	2.55	
	∠sn_ch_pg_L	−2.81	3.55	−9.76	4.15		0.00	1.60	−3.13	3.13		−2.81	3.63	−9.92	4.30	
	∠sn_ch_pg_R	−2.06	3.89	−9.70	5.57		−0.49	2.15	−4.71	3.73		−1.58	4.10	−9.62	6.47	
	∠g_n_prn	−3.15	4.13	−11.25	4.95		0.09	1.65	−3.14	3.32		−3.24	3.57	−10.24	3.77	
	∠g_prn_pg	−0.49	3.10	−6.57	5.58		−0.10	0.77	−1.60	1.40		−0.39	3.33	−6.91	6.12	
	∠cm_sn_ls	−4.80	2.33	−9.36	−0.24	<0.002*	−1.09	1.45	−3.93	1.75		−3.71	2.39	−8.39	0.98	<0.002*
	∠li_sm_pg	−4.95	2.06	−8.98	−0.92		0.02	2.33	−4.55	4.60		−4.98	2.82	−10.51	0.56	

*DA* Direct Anthropometry, *SGI* Smartphone generated 3D facial image, *mm* millimetre, ° Degrees, *SD* Standard Deviation, *LL* Lower Limit, *UL* Upper Limit

*p < 0.002, considered statistically significant

The findings of the method validity assessments are summarized in Table 5. The mean DDs between DA and SGI were generally negative for most measurements, accounting for 19 out of 32 measurements (> 58%), indicating that SGI had slightly higher measurement values compared to DA. Additionally, a significant difference (*p* < 0.002) in DDs for 9 out of 32 measurements (≈28%) suggested systematic bias between the DA and SGI methods, although the bias was generally small (± 0.2 mm). The ADs ranged from 0.72 to 1.87 mm for linear measurements and 3.83º to 5.00º for angular measurements, with the highest AD observed for "sn_gn" (1.87 mm) and "∠li_sm_pg" (5.00º). Similarly, when computing DDs between 3dMD and SGI, the mean DDs were overall negative since 12 of the SGI measures (> 37%) had higher measurement values than 3dMD. A small systematic bias (± 0.2 mm) was observed between the 3dMD and SGI methods, with 5 out of 32 measurements (≈16%) demonstrating significant DDs (*p* < 0.002). The ADs ranged from 0.47 to 1.72 mm for linear measurements and 1.21º to 1.66º for angular measurements, with the highest AD found for "ac_prn_R" (1.72 mm) and "∠ch_sn_ch" (1.66º). Furthermore, the mean DDs were generally negative, with 24 of the 3dMD measurements (75%) having somewhat higher measurement values compared to the DA method. Seven out of 32 measurements (≈22%) exhibited significant DDs (*p* < 0.002), indicating a systematic bias between the DA and 3dMD methods, which was generally small (± 0.2 mm). The ADs for linear and angular measurements ranged from 0.54 to 2.13 mm and 3.18º to 5.66º, respectively, with the highest AD observed for "n_sto" (2.13 mm) and "∠li_sm_pg" (5.66º).

**Table 5:** Method validity assessment

		DA-SGI				3dMD-SGI				DA-3dMD			
		**DD**		**SDD**	**AD**	**DD**		**SDD**	**AD**	**DD**		**SDD**	**AD**
Measurements (mm, °)		Mean ± SD	p*		Mean ± SD	Mean ± SD	p*		Mean ± SD	Mean ± SD	p*		Mean ± SD
Linear	al_al	1.20 ± 1.14	<0.002*	0.28	1.34 ± 0.97	1.40 ± 0.93	<0.002*	0.33	1.40 ± 0.93	−0.20 ± 0.86		−0.05	0.71 ± 0.49
	al_prn_L	1.18 ± 1.13	<0.002*	0.39	1.24 ± 1.06	0.40 ± 0.79		0.13	0.62 ± 0.62	0.78 ± 0.94		0.28	1.01 ± 0.68
	al_prn_R	1.40 ± 1.17	<0.002*	0.48	1.48 ± 1.06	0.92 ± 1.24		0.32	1.31 ± 0.78	0.48 ± 0.61		0.16	0.61 ± 0.47
	ac_ac	−0.64 ± 1.67		−0.14	1.34 ± 1.15	0.68 ± 1.22		0.15	1.25 ± 0.57	−1.32 ± 1.28	<0.002*	−0.27	1.61 ± 0.85
	ac_prn_L	−0.20 ± 1.52		−0.05	1.29 ± 0.77	0.75 ± 0.91		0.20	0.92 ± 0.73	−0.95 ± 1.55		−0.28	1.45 ± 1.07
	ac_prn_R	0.75 ± 1.15		0.23	1.11 ± 0.79	1.42 ± 1.22	<0.002*	0.43	1.72 ± 0.68	−0.67 ± 1.48		−0.19	1.37 ± 0.82
	sbal_sbal	0.01 ± 1.05		0.00	0.78 ± 0.69	0.09 ± 1.02		0.02	0.78 ± 0.63	−0.08 ± 1.20		−0.02	0.98 ± 0.66
	sbal_sn_L	−0.15 ± 1.47		−0.05	1.10 ± 0.96	−0.18 ± 0.82		−0.06	0.66 ± 0.50	0.02 ± 1.28		0.01	0.91 ± 0.87
	sbal_sn_R	0.12 ± 1.33		0.05	0.97 ± 0.89	0.07 ± 0.55		0.03	0.47 ± 0.28	0.05 ± 1.21		0.02	0.89 ± 0.78
	sbal_cph_L	0.71 ± 1.27		0.39	1.07 ± 0.96	0.74 ± 1.20		0.40	0.85 ± 1.11	−0.02 ± 1.29		−0.01	0.87 ± 0.92
	sbal_cph_R	0.86 ± 1.48		0.31	1.31 ± 1.07	1.36 ± 0.91	<0.002*	0.49	1.50 ± 0.63	−0.50 ± 1.70		−0.21	1.51 ± 0.85
	cph_cph	0.19 ± 0.87		0.07	0.74 ± 0.47	0.14 ± 0.73		0.05	0.47 ± 0.57	0.05 ± 1.02		0.02	0.77 ± 0.65
	ls_sto	0.07 ± 1.51		0.02	1.17 ± 0.90	0.98 ± 1.11		0.31	1.23 ± 0.81	−0.91 ± 1.58		−0.30	1.45 ± 1.06
	sto_li	−0.16 ± 1.04		−0.06	0.72 ± 0.75	−0.35 ± 0.96		−0.12	0.76 ± 0.66	0.18 ± 0.70		0.07	0.54 ± 0.48
	sn_sto	1.10 ± 1.47		0.32	1.45 ± 1.10	1.37 ± 0.91	<0.002*	0.40	1.54 ± 0.56	−0.27 ± 1.29		−0.08	1.06 ± 0.74
	sto_gn	−1.52 ± 1.64	<0.002*	−0.47	1.73 ± 1.40	−0.94 ± 1.23		−0.29	1.16 ± 1.01	−0.59 ± 1.48		−0.17	1.05 ± 1.18
	ch_ch	0.78 ± 1.74		0.12	1.59 ± 0.99	0.69 ± 1.41		0.11	1.15 ± 1.04	0.08 ± 1.36		0.01	1.13 ± 0.71
	n_sto	−0.97 ± 1.87		−0.12	1.76 ± 1.11	0.71 ± 1.07		0.09	1.07 ± 0.67	−1.68 ± 1.97		−0.19	2.13 ± 1.43
	sn_gn	−0.81 ± 2.04		−0.18	1.87 ± 1.07	−0.49 ± 1.13		−0.11	0.78 ± 0.94	−0.32 ± 1.98		−0.07	1.60 ± 1.16
	tr_sn	−0.70 ± 1.81		−0.08	1.57 ± 1.08	−0.89 ± 1.54		−0.10	1.48 ± 0.94	0.19 ± 1.30		0.02	0.86 ± 0.97
	n_sn	−1.54 ± 1.66	<0.002*	−0.23	1.84 ± 1.28	−0.20 ± 1.28		−0.03	0.84 ± 0.97	−1.34 ± 1.42	<0.002*	−0.19	1.62 ± 1.06
	sn_prn	0.23 ± 1.72		0.09	1.39 ± 0.98	1.02 ± 1.02	<0.002*	0.38	1.10 ± 0.92	−0.79 ± 1.30		−0.30	1.19 ± 0.92
Angular	∠tri_ch_pg_L	−3.66 ± 2.18	<0.002*	−0.70	3.83 ± 1.85	−0.91 ± 1.16		−0.17	1.21 ± 0.81	−2.75 ± 2.45	<0.002*	−0.52	3.18 ± 1.82
	∠tri_ch_pg_R	−2.84 ± 3.17		−0.40	3.77 ± 1.88	−0.95 ± 1.92		−0.14	1.55 ± 1.45	−1.89 ± 2.97		−0.29	2.98 ± 1.79
	∠ch_sn_ch	−4.49 ± 2.12	<0.002*	−0.60	4.56 ± 1.96	−1.04 ± 1.98		−0.14	1.66 ± 1.45	−3.45 ± 2.46	<0.002*	−0.49	3.84 ± 1.75
	∠ch_pg_ch	−2.50 ± 3.91		−0.31	4.23 ± 1.71	1.12 ± 2.07		0.14	1.54 ± 1.77	−3.62 ± 3.15	<0.002*	−0.48	4.04 ± 2.56
	∠sn_ch_pg_L	−2.81 ± 3.55		−0.39	3.92 ± 2.15	0.00 ± 1.6		0.00	1.25 ± 0.95	−2.81 ± 3.63		−0.43	4.11 ± 1.89
	∠sn_ch_pg_R	−2.06 ± 3.89		−0.23	3.68 ± 2.31	−0.49 ± 2.15		−0.05	1.51 ± 1.57	−1.58 ± 4.10		−0.18	3.73 ± 2.17
	∠g_n_prn	−3.15 ± 4.13		−0.35	4.83 ± 1.68	0.09 ± 1.65		0.01	0.92 ± 1.35	−3.24 ± 3.57		−0.37	4.45 ± 1.70
	∠g_prn_pg	−0.49 ± 3.10		−0.15	2.46 ± 1.85	−0.10 ± 0.77		−0.03	0.58 ± 0.49	−0.39 ± 3.33		−0.11	2.56 ± 2.07
	∠cm_sn_ls	−4.80 ± 2.33	<0.002*	−0.42	4.80 ± 2.33	−1.09 ± 1.45		−0.10	1.09 ± 1.45	−3.71 ± 2.39	<0.002*	−0.31	3.79 ± 2.25
	∠li_sm_pg	−4.95 ± 2.06	<0.002*	−0.56	5.00 ± 1.93	0.02 ± 2.33		0.00	1.32 ± 1.90	−4.98 ± 2.82	<0.002*	−0.58	5.66 ± 0.51

*DA* Direct Anthropometry, *SGI* Smartphone generated 3D facial image, *DD* Directional Difference, *SDD* Standardized Directional Difference, *AD* Absolute Difference, *mm* millimetre, ° Degrees, *SD* Standard Deviation

*p < 0.002 (in** bold **italics), considered statistically significant

Table 6 illustrates a comparison of MADs between different methods. DA-SGI had the highest MAD for both linear measurements (1.31 ± 0.34 mm) and angular measurements (4.11 ± 0.76º), while 3dMD-SGI displayed the lowest MAD for both linear measurements (1.05 ± 0.36 mm) and angular measurements (1.26 ± 0.33º).

**Table 6:** Mean absolute differences between different methods

	**MAD**		
	**DA-SGI**	**3dMD-SGI**	**DA-3dMD**
**Measurements**	Mean ± SD	Mean ± SD	Mean ± SD
**Linear (mm)**	1.31 ± 0.34	1.05 ± 0.36	1.15 ± 0.40
**Angular (°)**	4.11 ± 0.76	1.26 ± 0.33	3.83 ± 0.86

*MAD* Mean absolute difference, *DA* Direct Anthropometry, *SGI* Smartphone generated 3D facial image, *mm* millimetre, ° Degrees, *SD* Standard Deviation

## Discussion

The reliability of 3D surface imaging systems has been explored by researchers to identify a viable system for capturing 3D images in clinical and research contexts [58–61]. With the introduction of handheld, versatile, and affordable scanning devices, the range of potential applications [62–64] has expanded, including their use for quantification and objective assessment of CLP deformity [27, 28]. The sector is continuously advancing, with new systems frequently being presented to the market. However, before incorporating these systems into routine clinical settings, their validity needs to be established to assess their performance against our current anthropometry practice and their acceptability for usage in patients. Therefore, this study attempted to evaluate the validity of SGI for routine clinical application in assessing the oronasal region of patients with cleft by comparing the linear and angular facial measurements acquired from SGI with those obtained from DA and 3dMD-generated images.

The 3dMD is widely considered the gold standard for 3D surface imaging [61, 65–67] due to its precision, reproducibility, and accuracy, with an average technical error of 0.35 ± 0.14 mm [64] and a mean global error of 0.2 mm [68]. However, some studies have also suggested DA as a gold standard [1, 55, 69, 70]. Therefore, for the precise validity assessment of SGI, this study compared smartphone photogrammetry with both 3dMD and DA. Previous research by *Liu *et al*.* assessed the accuracy of 3D stereophotogrammetry by comparing *Bellus3D Face Camera Pro*, an Android-based universal serial bus (USB) camera, with 3dMD and DA [70]. In this study, *Bellus3D FaceApp*, which employs the iPhone or iPad's built-in TrueDepth camera, was utilized to generate high-resolution 3D facial scans without the need for an auxiliary camera. The quality of the 3D images generated by *Bellus3D FaceApp*, particularly the triangular mesh reflecting the surface, has been reported to be higher compared to other face scanning applications [42, 71] which was essential for the analysis of 3D images of the oronasal region in this work.

**Fig. 4:**
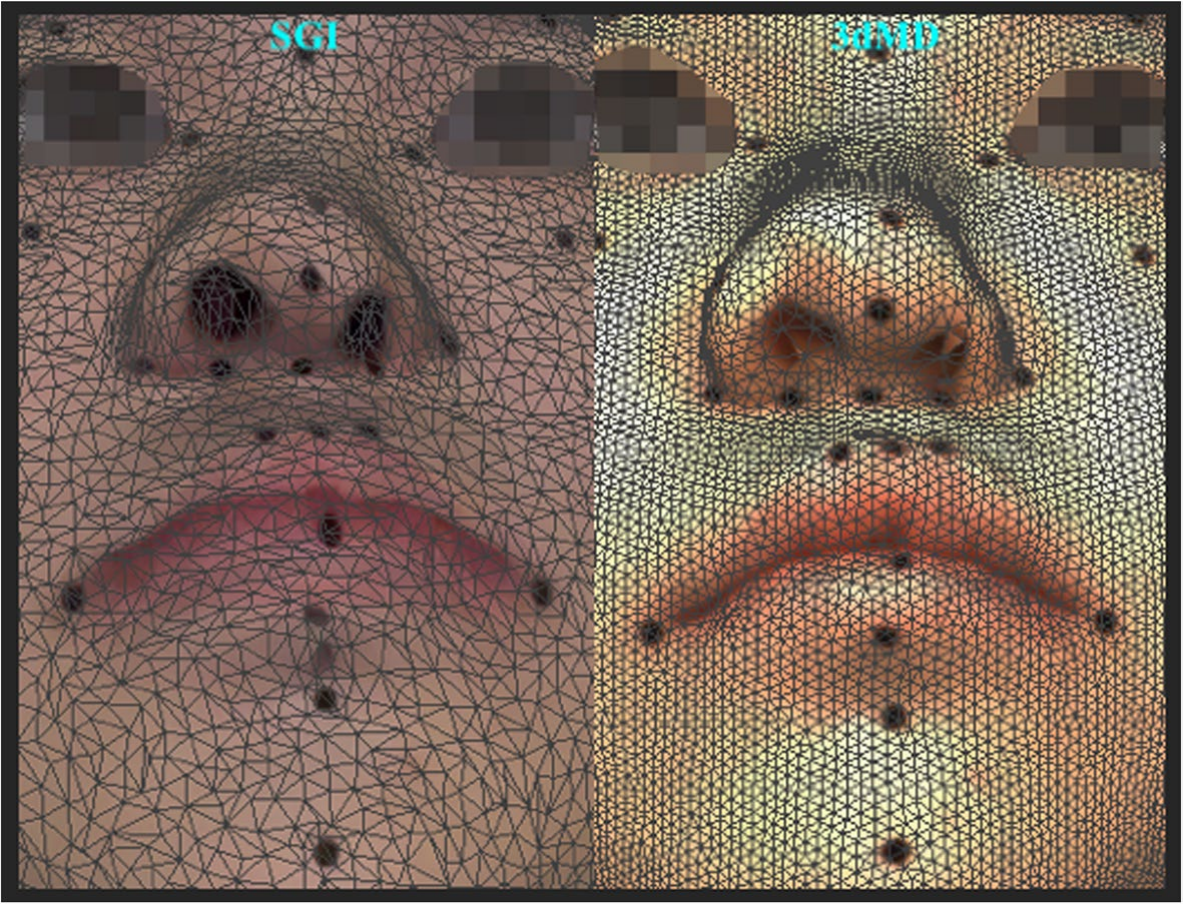
An illustrative image of the oronasal region showcasing the variances in inter-vertex distances and the distribution of triangles in the polygon mesh of SGI and 3dMD rendered 3D facial image

The current study evaluated both linear and angular measurement methods in the validity assessment, as clinically validated objective assessments are often regarded as the benchmark for measuring outcomes and are more representative of the clinical setting than the landmark coordinate approach [72]. To achieve this, the current investigation included multiple landmarks for inter-landmark measurements. While certain landmarks were easily identifiable due to distinct borders, others were located on curved areas of the face and required palpation for accurate identification. Since the identification of anatomic landmarks is subjective and relies on factors such as anatomical structure, colour, and reflection [73], *Aynechi *et al*.* advocated labelling landmarks before facial scanning [51]. Therefore, in this work, all landmarks were labelled before image acquisition to enhance the accuracy and reproducibility of the measures [51, 74]. Although there were no noticeable differences between the DA, SGI, and 3dMD methods in terms of inter-landmark linear and angular measures, the 3D digital measurements generally had higher values than the DA, which accords with previous study findings [1, 51, 75]. Specifically, there was a trend towards higher inter-landmark distances in terms of DD and AD with SGI compared to 3dMD and DA. This disparity can be explained by the longer scanning time required by *Bellus3D FaceApp* (10 s) compared to 3dMD (≈1.5 ms), which may have introduced errors and motion artefacts due to involuntary facial and head movements during scanning [3, 76]. Another factor that could have affected the resolution, aesthetic rendering, and accuracy of the SGI method [77] would be the presence of higher inter-vertex distances or sparsely dispersed triangles in the polygon mesh of SGI (Fig. 4).

Disparities between face acquisition systems or between a face acquisition system and DA of 1–3 mm are deemed clinically acceptable, according to prior research. The acceptable deviation limits differ among studies, with some viewing deviations of less than 1 mm as acceptable [42, 70], while others define deviations of less than 2 mm as reliable [1, 78, 79]. However, recent investigations reinforced the assumption that a considerable deviation of 3 mm or less is only clinically relevant for extreme, thorough evaluations of micro-aesthetics [36, 39, 80]. In the context of routine clinical applications such as orthodontics, prosthodontics, and maxillo-facial surgery requiring digital landmark annotation, 3D modelling, treatment simulation, and patient education, deviations of 3 mm or less are clinically irrelevant and can be deemed acceptable. Therefore, a 95% limit of agreement beyond 3 mm was considered clinically unacceptable for linear measurements in the current investigation. Additionally, for angular measurements, deviations beyond 5º were considered clinically unacceptable [55]. Most of the measurements in the study showed clinically acceptable differences and good agreement between SGI, DA, and 3dMD. The accuracy of SGI can be deemed somewhat comparable to 3dMD but inferior to DA, as 59% of the measurements between 3dMD and SGI fell within acceptable limits, compared to 16% for DA and SGI. It is worth mentioning that the percentage of measurements with clinically acceptable differences was high when deviations beyond 3 mm and 5º were considered unreliable. This fraction would have fallen if the acceptable criteria were set to 2 mm and 4º or 1 mm and 3º. Thus suggesting that SGI may not be beneficial for detailed evaluations such as virtual treatment planning, virtual articulation or airway analysis in CLP cases with obstructive sleep apnea (OSA).

Trueness in this study was operationalized as the accuracy of a set of measurements in relation to a reference value established by DA and 3dMD. To evaluate trueness, we compared the MAD of SGI with the gold standard DA and with 3dMD, a widely accepted gold standard in stereophotogrammetry. SGI demonstrated reasonable trueness with a MAD of 1.31 ± 0.34 mm for linear measurements and 4.11 ± 0.76º for angular measurements compared to DA, and 1.05 ± 0.36 mm for linear measurements and 1.26 ± 0.33º for angular measurements compared to 3dMD. The plausibility of the trueness values of SGI was supported by the MAD values of 1.15 ± 0.40 mm and 3.83 ± 0.86º for linear and angular measures, respectively, observed in the direct comparison between DA and 3dMD. These MAD values were closely aligned with the trueness values exhibited by SGI. However, a recent study by *Liu *et al. reported smaller trueness values between *Bellus3D* and DA, with 0.61 ± 0.47 mm for linear measurements and 0.99 ± 0.61º for angular measurements, as well as between *Bellus3D* and 3dMD, with 0.38 ± 0.37 mm for linear measurements and 0.62 ± 0.39º for angular measurements. The disparity in trueness values can be elucidated by disparities in research configurations. *Liu *et al*.* utilized *Bellus3D Face Camera Pro*, which captures over 500,000 3D facial data points [81] of the user's face. Nevertheless, it is crucial to acknowledge that their study used a mannequin head, which lacks the complex 3D configuration of the human face, including its convexities, concavities, and intricate angles. Using a mannequin head eliminates the influence of soft tissue drape, which can significantly impact the positioning and measurement of landmarks [82]. The current study employed the iPhone's built-in TrueDepth camera-based *Bellus3D FaceApp*, which captures fewer, around 250,000 3D data points, of real patients' faces, reflecting the true clinical situation. Hence, it is plausible that the limited quantity of data points captured in our study may have played a role in the elevated trueness values.

**Fig. 5:**
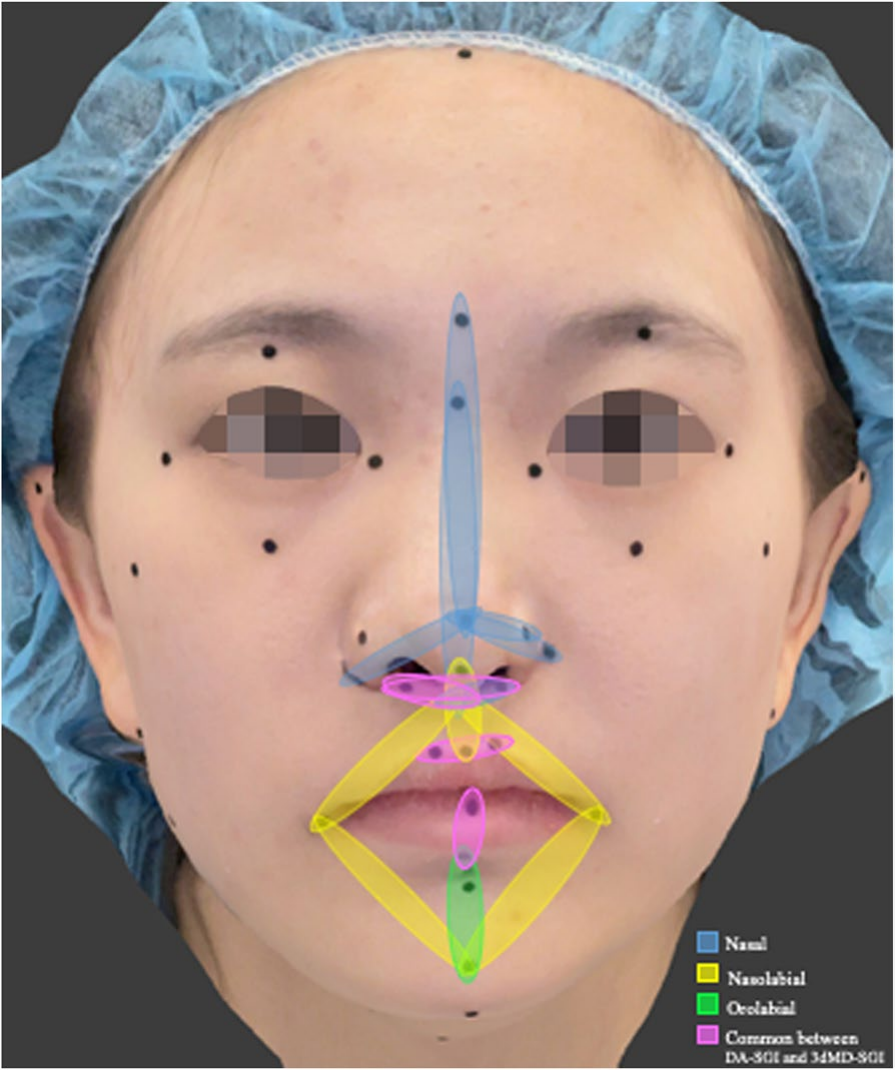
A visual representation demonstrating the accuracy of SGI in various oronasal areas. The accurate measures in the nasal area are indicated by blue, the nasolabial area by yellow, and the orolabial area by green. The accurate measures common between the DA-SGI and 3dMD-SGI methods are highlighted in pink

Given that the oronasal region is the most clinically significant craniofacial area affected in patients with CLP, we performed an area-wise assessment across SGI, DA, and 3dMD specifically focusing on the oronasal region and analysed inter-landmark measures specific to the nasal, nasolabial, and orolabial areas and their adjacent soft tissue landmarks to establish the most accurate area of the oronasal region. The findings indicated that the orolabial area of SGI's oronasal region was more accurate compared to the nasal and nasolabial areas. Within the orolabial area, 50% of the measures (averaged across DA-SGI and 3dMD-SGI) demonstrated clinically acceptable differences compared to 41.5% and 28.5% measures within the nasal and nasolabial areas, respectively. More specifically, the width of the philtrum, vermilion height of the lower lip, and labiale inferius showed the smallest clinically acceptable differences [DA-SGI (lower limit, LL to upper limit, UL): cph_cph, −1.52 to 1.90 mm; sto_li, −2.20 to 1.87 mm; 3dMD-SGI (LL to UL): cph_cph, −1.29 to 1.57 mm; sto_li, −2.23 to 1.53 mm; ∠li_sm_pg, −4.45º to 4.60º, Table 4] within the orolabial area. Likewise, the subalare width and subnasale [sbal_sbal, −2.05 to 2.08 mm and −1.90 to 2.09 mm; and sbal_sn (right), −2.49 to 2.72 mm and −1.02 to 1.16 mm in DA-SGI and 3dMD-SGI, respectively, Table 4) in the nasal area and the columella, subnasale, labiale superius, and cheilion [3dMD-SGI (LL to UL): ∠cm_sn_ls, −3.93º to 1.75º; ∠ch_sn_ch, −4.92º to −2.83º; Table 4] in the nasolabial area with clinically acceptable differences were found to be more accurate. These results were in agreement with *Othman *et al*.*’s findings [1]. Besides, some of the soft tissue landmarks, such as the nasion and gnathion [3dMD-SGI (LL to UL): n_sto, −1.38 to 2.79 mm; sn_gn, −2.71 to 1.73 mm, Table 4] adjacent to the nasal, nasolabial, and orolabial areas also exhibited the smallest clinically acceptable difference and were found to be accurate. A visual depiction of the accuracy of SGI in various oronasal areas has been presented in Fig. 5.

Previous research has employed 3D surface comparison to evaluate flat and curved areas [42] and central and lateral areas [83, 84] of the face. The present study went one step further and analysed the oronasal region in terms of central, paracentral, and lateral areas, as well as flat, prominent, and concave areas specific to this region, across SGI, DA, and 3dMD. Our results showed that the central oronasal areas in SGI, including the vermilion height of the lower lip, width of the philtrum, subalare, subnasale, columella, and labiale superius, with the smallest clinically acceptable difference [DA-SGI (LL to UL): sto_li, −2.20 to 1.87 mm; cph_cph, −1.52 to 1.90 mm; 3dMD-SGI (LL to UL): sbal-sn, −1.79 to 1.43 mm (left) and −1.02 to 1.16 mm (right); cph_cph, −1.29 to 1.57 mm; ∠cm_sn_ls, −3.93º to 1.75º, Table 4] was more accurate compared to the paracentral and lateral oronasal areas. This finding was consistent with a prior study conducted by *Gallardo *et al*.* that reported major deviations in the lateral region of the face compared to the central region [83]. Furthermore, the flat areas of the oronasal region in SGI (averaged across DA-SGI and 3dMD-SGI), particularly the subalare, subnasale, and cheilion with the smallest clinically acceptable difference [DA-SGI (LL to UL): sbal-sn (right), −2.49 to 2.72 mm; 3dMD-SGI (LL to UL): sbal-sn, −1.79 to 1.43 mm (left) and −1.02 to 1.16 mm (right); ∠ch_sn_ch, −4.92º to −2.83º, Table 4] were more accurate compared to the prominent or concave areas, in agreement with the findings of *D'Ettorre *et al*.* [42]. *Bellus3D's* image stitching technique could be ascribed to the good reliability of SGI in the central and flat oronasal regions. This technique combines 3D point clouds acquired from the moving head of the subject to create a composite 3D image. The stitching alignment is dependent on the facial features, and it works well for the central and flat oronasal regions. However, for prominent or laterally located landmarks, the accuracy of reconstruction utilizing this technique is limited. To improve the accuracy of the generated 3D facial image, markers can be placed on the lateral oronasal area for precise alignment and subsequent stitching [85]. An area-wise assortment of the SGI’s accuracy in the oronasal region based on the landmarks common between the DA-SGI and 3dMD-SGI methods is illustrated in Table 7.

**Table 7:**
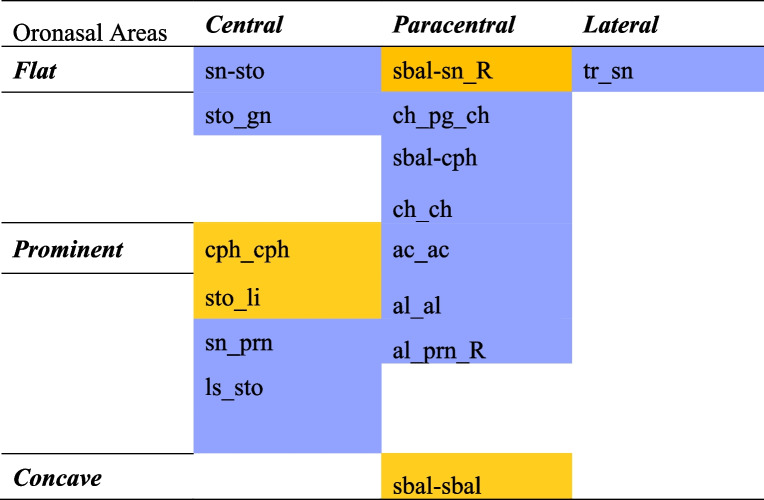
Area-wise assortment of SGI accuracy in the oronasal region^a^

Accurate (≤ 3 mm or ≤ 5º),  Inaccurate (> 3 mm or > 5º)

*DA* Direct Anthropometry, *SGI* Smartphone generated 3D facial image

^a^Only common landmarks between DA-SGI and 3dMD-SGI methods have been represented

As for the least accurate region, the nasolabial area was found to be the least accurate as around 28.5% of the measures (averaged between DA-SGI and 3dMD-SGI) demonstrated clinically unacceptable differences. This could be attributed to the distortion of the soft tissues in the nasolabial area caused by the surgical scars between the subalare and crista philtri (sbal_cph) or between the subnasale and stomion (sn_sto), potentially leading to measurement inaccuracies. Additionally, the "anatomical width of the nose," which involved using the "alar crest," a nasal area landmark, exhibited the highest clinically acceptable differences in DA-SGI [ac_ac (LL to UL), −3.92 to 2.64 mm] as well as 3dMD-SGI [ac_ac (LL to UL), −1.71 to 3.07 mm, Table 4] and was the most inaccurate measure. The prominent contour and lack of rigidity of the ‘alar crest’ pose challenges in precisely placing the calliper point during DA measurements, which may have further contributed to the inaccuracies observed. In summary, the findings showed that SGI's accuracy was higher in the orolabial area and certain specific central and flat areas within the oronasal region. Thus, making it suitable for assessing the philtrum width, lower lip vermilion, subalare width, and nasolabial angle in the oronasal region. However, it may not be accurate enough for tasks that are critical for clinical application in CLP cases, such as comprehensive assessment of the oronasal morphology, virtual treatment planning, virtual articulation, and airway analysis in patients with OSA. Indeed, from the standpoint of clinical application, SGI’s accuracy in encompassing the whole oronasal region would be ideal.

Notwithstanding the thorough examination, it is important to take into account certain limitations for this study. The study may be constrained by the possibility of patient movement during image acquisition that could have introduced motion artefacts. Even though *Bellus3D FaceApp* is a static scanning system, it necessitates the participant to move their head, thus potentially affecting the position of their neck muscles and introducing inaccuracies. Moreover, we exclusively assessed adult participants who were compliant and anticipated to sustain the necessary head-face position with minor involuntary movements. Consequently, the outcomes may not be applicable to young or uncooperative individuals. Another limitation is the potential clinical applicability constraints of SGI for pre-surgical evaluation before lip repair. While SGI demonstrated fair trueness compared to DA and 3dMD, it may not offer the necessary accuracy required for precise measurements and detailed assessment of the oronasal region crucial for surgical planning. Additionally, capturing such images in young cleft patients, particularly before lip repair typically performed in children, may be challenging due to difficulties in maintaining the necessary head-face position with minimal involuntary movements. Furthermore, we used the Apple iPhone 12 (iOS 14.8.1) to capture the images. It is worth mentioning that the type of smartphone's operating system (Android or iOS-based) and, to some extent, its version might have an impact on the accuracy of SGI. Upgrading phone models with higher-resolution cameras and improved hardware and software features can enhance the accuracy of the SGI. Lastly, *Bellus3D Inc.* has ceased its 3D face scanning operations recently; however, the results of this study could aid in the creation of a more sophisticated and affordable 3D face-acquisition system.

Several studies have examined the accuracy of 3D face-acquisition systems. However, most of the face-acquisition systems currently on the market are expensive, and their use may not be warranted for regular clinical purposes. Conversely, low-cost systems like *Bellus3D FaceApp* may not offer the necessary level of accuracy for clinical purposes. Nevertheless, they could still be useful in patients with CLP for macroscopic oronasal analysis, as well as for automated landmark detection, machine learning, or simulating treatments to aid patient learning, motivation, and communication. Future studies aiming to leverage smartphone-based 3D face acquisition for analyzing the oronasal region in CLP cases could focus on automated or semi-automatic markers on the lateral oronasal area for alignment. This can be followed by algorithm-based stitching to achieve wider precision. Furthermore, researchers could explore the application of surface-based methods to compare SGI with 3dMD images, allowing for a comprehensive analysis of shape differences and surface details between these two 3D imaging modalities. Additionally, future research should investigate the impact of variables such as soft tissue scars, deep grooves, or hair in the oronasal region and lighting on image quality, as these factors have been known to cause image distortions and artefacts [86, 87]. As smartphone technology and applications continue to advance, we can anticipate improved precision and quality in smartphone-based 3D face acquisition, thereby enhancing the potential clinical use of SGI in assessing the oronasal region.

## Conclusions

The study yielded the following conclusions:

1. The DA, SGI, and 3dMD methods demonstrated no statistically significant difference in their inter-landmark linear and angular measures. Additionally, there was good agreement across SGI, DA, and 3dMD, with the majority of measures exhibiting clinically acceptable variation in differences.
2. SGI displayed fair trueness, with values of 1.31 ± 0.34 mm and 4.11 ± 0.76º compared to DA, and 1.05 ± 0.36 mm and 1.26 ± 0.33º compared to 3dMD.
3. The orolabial area and certain specific central and flat areas within the oronasal region of SGI in patients with CLP exhibit high accuracy, outperforming the nasal, nasolabial, praracentral, lateral, prominent, and concave oronasal areas.
4. The results suggest that SGI has limited clinical applicability for assessing the entire oronasal region of patients with CLP and that SGI’s accuracy in encompassing the whole oronasal region would be ideal for optimal clinical use. However, SGI could still be valuable for macroscopic oronasal analysis or for treatment simulations to aid patient education, where accuracy within 3 mm and 5º may not be critical.

## Supplementary Information

Supplementary Material 1

## Data Availability

The datasets used and/or analysed during the current study are available from the corresponding author on reasonable request.

## Abbreviations

CLP: Cleft lip and palate
2D: Two-dimensional
3D: Three-dimensional
SGI: Smartphone-generated 3D facial images
DA: Direct anthropometry
CL: Cleft lip
ICC: Intraclass correlation coefficient
NHP: Natural head position
MIP: Maximum intercuspation position
HD: High definition
LED: Light-emitting diode
App: Application
OBJ: Object file
SD: Standard deviation
MAD: Mean absolute difference
ANOVA: A one-way analysis of variance
SPSS: Statistical Package for the Social Sciences
DD: Directional differences
SDD: Standardised directional differences
AD: Absolute differences
USB: Universal serial bus
OSA: Obstructive sleep apnea
LL: Lower limit
UL: Upper limit
