# Supplementary material for: Smartphone-generated 3D facial images: reliable for routine assessment of the oronasal region of patients with cleft or mere convenience? A validation study
Source: BMC Oral Health. 2024 Dec 19;24:1517. doi: 10.1186/s12903-024-05280-9 (PMC11660614; doi:10.1186/s12903-024-05280-9)
Supplement: Supplementary file 1 — Supplementary Material 1 [file 12903_2024_5280_MOESM1_ESM.docx]

**Supplementary Appendix 1**. Intra-assessor reliability and method error between measurements

|  |  | SGI | | | 3dMD | | |
| --- | --- | --- | --- | --- | --- | --- | --- |
| Measurements (mm, °) | | ICC | CI | DE | ICC | CI | DE |
| Linear | al_al | 0.99 | 0.99 to 1.00 | 0.08 | 0.99 | 0.98 to 1.00 | 0.08 |
|  | al_prn_L | 0.98 | 0.90 to 0.99 | 0.13 | 0.99 | 0.97 to 1.00 | 0.08 |
|  | al_prn_R | 1.00 | 0.99 to 1.00 | 0.04 | 0.96 | 0.89 to 0.99 | 0.15 |
|  | ac_ac | 1.00 | 0.99 to 1.00 | 0.09 | 0.96 | 0.86 to 0.99 | 0.29 |
|  | ac_prn_L | 0.99 | 0.97 to 1.00 | 0.10 | 0.99 | 0.98 to 1.00 | 0.09 |
|  | ac_prn_R | 1.00 | 1.00 to 1.00 | 0.04 | 0.97 | 0.92 to 0.99 | 0.17 |
|  | sbal_sbal | 1.00 | 0.99 to 1.00 | 0.06 | 0.99 | 0.97 to 1.00 | 0.10 |
|  | sbal_sn_L | 0.99 | 0.96 to 1.00 | 0.07 | 0.97 | 0.90 to 0.99 | 0.12 |
|  | sbal_sn_R | 0.96 | 0.90 to 0.99 | 0.12 | 0.98 | 0.93 to 0.99 | 0.11 |
|  | sbal_cph_L | 0.99 | 0.98 to 1.00 | 0.04 | 0.94 | 0.82 to 0.98 | 0.08 |
|  | sbal_cph_R | 0.99 | 0.98 to 1.00 | 0.07 | 0.96 | 0.87 to 0.99 | 0.15 |
|  | cph_cph | 1.00 | 0.99 to 1.00 | 0.05 | 1.00 | 0.99 to 1.00 | 0.04 |
|  | ls_sto | 0.99 | 0.97 to 1.00 | 0.08 | 0.98 | 0.91 to 1.00 | 0.10 |
|  | sto_li | 0.99 | 0.98 to 1.00 | 0.06 | 0.99 | 0.97 to 1.00 | 0.07 |
|  | sn_sto | 0.99 | 0.96 to 1.00 | 0.09 | 0.99 | 0.98 to 1.00 | 0.09 |
|  | sto_gn | 0.97 | 0.90 to 0.99 | 0.14 | 0.99 | 0.96 to 1.00 | 0.09 |
|  | ch_ch | 1.00 | 0.99 to 1.00 | 0.10 | 0.98 | 0.94 to 0.99 | 0.13 |
|  | n_sto | 1.00 | 1.00 to 1.00 | 0.06 | 1.00 | 1.00 to 1.00 | 0.09 |
|  | sn_gn | 0.99 | 0.96 to 1.00 | 0.14 | 0.99 | 0.97 to 1.00 | 0.14 |
|  | tr_sn | 1.00 | 1.00 to 1.00 | 0.08 | 1.00 | 1.00 to 1.00 | 0.07 |
|  | n_sn | 1.00 | 0.99 to 1.00 | 0.11 | 1.00 | 0.99 to 1.00 | 0.11 |
|  | sn_prn | 0.98 | 0.93 to 0.99 | 0.08 | 0.99 | 0.98 to 1.00 | 0.06 |
| Angular | ∠tri_ch_pg_L | 0.95 | 0.86 to 0.98 | 0.20 | 0.99 | 0.98 to 1.00 | 0.12 |
|  | ∠tri_ch_pg_R | 1.00 | 0.99 to 1.00 | 0.11 | 1.00 | 0.99 to 1.00 | 0.10 |
|  | ∠ch_sn_ch | 0.99 | 0.98 to 1.00 | 0.19 | 0.99 | 0.97 to 1.00 | 0.20 |
|  | ∠ch_pg_ch | 1.00 | 0.99 to 1.00 | 0.13 | 0.99 | 0.98 to 1.00 | 0.14 |
|  | ∠sn_ch_pg_L | 0.99 | 0.97 to 1.00 | 0.21 | 0.99 | 0.96 to 1.00 | 0.19 |
|  | ∠sn_ch_pg_R | 0.99 | 0.96 to 1.00 | 0.18 | 1.00 | 1.00 to 1.00 | 0.10 |
|  | ∠g_n_prn | 1.00 | 1.00 to 1.00 | 0.12 | 1.00 | 1.00 to 1.00 | 0.06 |
|  | ∠g_prn_pg | 1.00 | 1.00 to 1.00 | 0.03 | 1.00 | 1.00 to 1.00 | 0.02 |
|  | ∠cm_sn_ls | 1.00 | 0.99 to 1.00 | 0.19 | 0.99 | 0.98 to 1.00 | 0.22 |
|  | ∠li_sm_pg | 1.00 | 0.97 to 1.00 | 0.17 | 1.00 | 0.99 to 1.00 | 0.16 |

SGI, Smartphone generated 3D facial image; mm, millimetre; °, Degrees; ICC, Intra-class correlation coefficient; CI, 95% Confidence Interval; DE, Dahlberg’s Error
